# A 100-Mhz Bandwidth 80-dB Dynamic Range Continuous-Time Delta-Sigma Modulator with a 2.4-Ghz Clock Rate

**DOI:** 10.1186/s11671-020-3284-4

**Published:** 2020-03-06

**Authors:** Yao Xiao, Zhifei Lu, Zhaofeng Ren, Xizhu Peng, He Tang

**Affiliations:** School of Electronic Science and Engineering, SoC Design Center, University of Electronic Science and Technology of China, Chengdu, 610054 China

**Keywords:** Delta-sigma modulator, Wide-bandwidth, Nanoscale CMOS process, Continuous-time

## Abstract

The bandwidth of a *ΔΣ* modulator is limited by the clock rate due to the oversampling ratio requirement. As the nanoscale CMOS processes are developing rapidly, it is possible to design wide bandwidth and high dynamic range continuous-time *ΔΣ* modulators for high-frequency applications. This paper proposes a 3rd-order 4-bit continuous-time *ΔΣ* modulator with a single-loop feedforward topology. This modulator is designed in a 40-nm CMOS process and achieves 80-dB dynamic range and a 100-MHz bandwidth at a clock rate of 2.4 GHz. The modulator consumes 69.7 mW from 1.2 V power supply.

## Introduction

Driven by the increasing demands in wireless communication applications such as cellular standards, analog-to-digital converters (ADCs) evolve rapidly to support higher signal bandwidth (BW) and dynamic range (DR). The requirement of BW in Long-Term-Evolution Advanced (LTE-A) communication standard has risen to 100 MHz. Nyquist ADCs, typically pipeline ADCs [1, 2], have been used in macro base stations for their high BW. However, indispensable input buffers for driving thermal-noise-limited switched input capacitors and anti-aliasing filter cause significant power consumption and design complexity. Furthermore, the fact that pipeline ADCs rely on accurate inter-stage gain, which determines high-gain wideband residue amplifier and calibration technology, leads to complexity and power dissipation. *ΔΣ* ADCs are known for their high performance and power efficiency employing oversampling and noise shaping technology. However, the requirement of oversampling ratios (OSRs), which is typically over 16 [3–6], determines sampling frequency beyond GHz. Recently, *ΔΣ* ADCs exceeding 50 MHz BW have been proposed by using nanoscale CMOS processes, which allow multi-GHz clock rate. Previously, high frequency *ΔΣ* ADCs usually adopt continuous-time (CT) realizations [3–9] instead of discrete-time (DT) realizations. The latter is implemented by switched capacitor circuit, and its accuracy relies on capacitor matching, which means a robust operation under process variation is offered. Besides, superior immunity to clock jitter is provided since the time constants of the capacitors and switches are sufficiently small. However, as the sampling operation executes before modulator, the anti-aliasing filter is needed. On the other hand, due to the settling requirement to ensure stability in the stages, operational amplifiers in DT modulators are implemented with broader unity-gain bandwidth (UGBW) than CT modulators. In summary, DT modulators can provide high accuracy but narrow signal [10, 11] and are widely used to implement metering applications such as smart sensors and biomedical imaging. In contrast, there has been more widespread effort to design CT modulators for high frequency applications than DT ones with comparable complexity and power consumption.

The demanding design target of higher BW in a given process determines a lower OSR because of process-limited clock rate. To achieve a sufficient DR, an aggressive noise shaping implemented by high noise transfer function order, which is conventionally performed by loop filter cascade and generally equal or greater than 3 in previous works, is required. However, the increased loop filter orders cause power consumption, instability, and design complexity. The multi-stage noise-shaping (MASH) architecture [6, 8], implemented by cascaded low-order local *ΔΣ* modulators without feedback path among each other, was employed to alleviate stability issues but with mismatch sensitiveness. Moreover, a modulator with a multi-bits quantizer gets a conditionally high DR with an exponential increasing comparator amount.

This paper describes a CT modulator in 40 nm CMOS that achieves 80 DR over 100 MHz BW with 69.7 mW consumption using 40 nm CMOS process. This paper is organized as follows. The “Method” section describes the modulator topology and circuit implementation. The “Results and Discussion” section shows simulated results, and the “Conclusion” section provides a summary of this work.

## Method

**Fig. 1:**
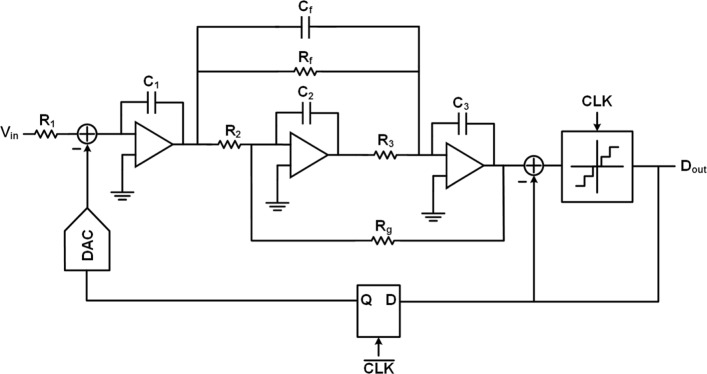
Overall schematic of proposed single-loop 3rd-order 4-bit CT *ΔΣ* modulator with a feedforward topology

Figure 1 illustrates the overall schematic of the proposed 3rd-order CT *ΔΣ* modulator with the single-ended structure for simplification. The 3rd-order noise shaping gets a great compromise between the DR and the loop stability. The proposed modulator has a sampling rate of 2.4 GHz with a 12 OSR. The relatively high OSR in *ΔΣ* modulators exceeded 100 MHz BW ensures a high DR. The modulator contains three RC integrators, a 4-bit quantizer and a 4-bit current-steering DAC. The integrators are implemented by innovational low-power dissipation feedforward amplifiers for high energy efficiency. The feedback DAC has a half sampling period duration extra delay to relax the metastability requirement of the quantizer. A fast feedback path implemented by a passive adder and driven directly by quantizer realizes the excess-loop-delay (ELD) compensation. A feedforward topology is employed for power efficiency at the expense of out-of-band signal-transfer-function.

### Amplifier Design

**Fig. 2:**
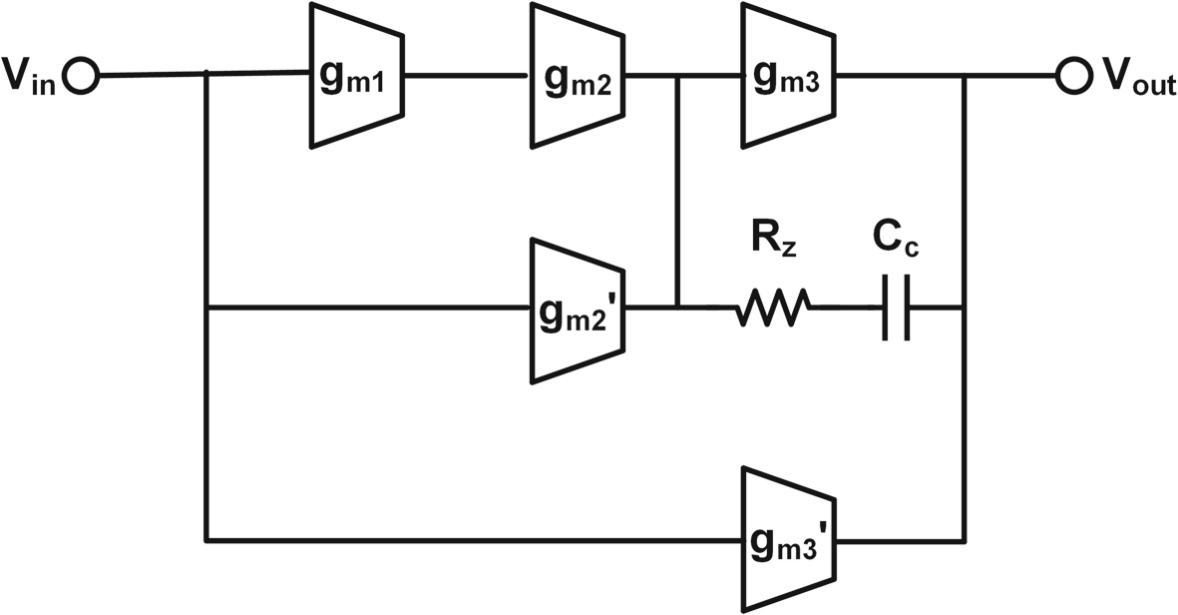
Topology of the proposed three-stage feedforward amplifier with Miller compensation

High amplifier gain is required in *ΔΣ* modulators to ensure the desired noise transform function. However, the nanoscale technologies used to achieve multi-GHz clock rate suffer in low intrinsic gain. Therefore, a three-stage amplifier is adopted to implement sufficient DC gain, as shown in Fig. 2. Feedforward topology and Miller compensation are combined to improve phase margin without unit gain bandwidth reduction. Feedforward amplifiers have been one popular solution of achieving high gain with adequate UGBW and phase margin (PM). The left-half plane zero caused by the feed-forward path is supposed to effectively cancel the negative phase shift of poles. It requires high transconductance of the amplifiers on the feed-forward path and consumes significant power. The advantaged scheme of re-using bias current saves power whereas it limits *g*_*m*_ values. Insufficient *g*_*m*_ typically causes the zero beyond the UGBW and cannot provide an adequate phase margin. An optimized zero located below the overall UGBW is provided by adding a Miller compensating capacitor and a nulling resistor.

**Fig. 3:**
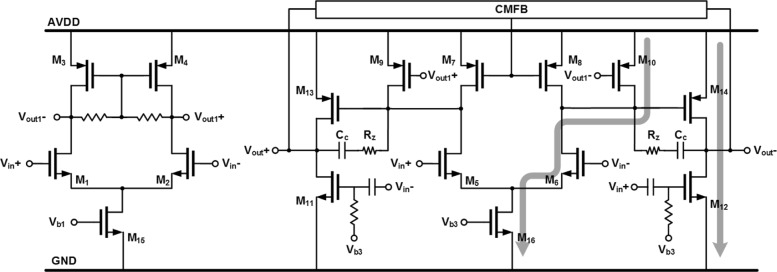
Amplifier transistor-level schematic

**Fig. 4:**
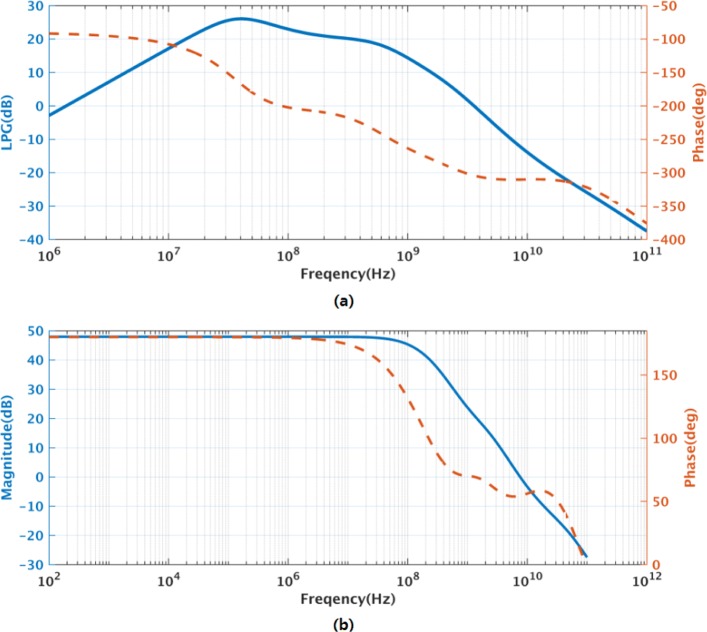
The post-layout simulated results of the amplifier in the 1st integrator. **a** Open-loop ac response; **b** Close-loop ac response

Figure 3 shows the transistor-level schematic of the amplifier used in the first integrator. Transistors *M*_1−4_ form the input stage of amplifier, while transistors *M*_9,10_ and *M*_13,14_ form the second and third stage, respectively. Transistors *M*_5−8_ and *M*_11,12_ create two high-speed feedforward paths between the input and output while sharing bias currents with the second- and third-stage amplifiers. The first-stage output common-mode (CM) is fixed locally. The second-stage and the 3rd-stage output CM is fixed by a second-stage feedback path across a CMFB amplifier, *M*_7,8_ and *M*_13,14_. Figure 4 a shows the simulated post-layout open-loop response of amplifier of the first integrator with all loading while Fig. 4 b shows the close-loop response. The first integrator achieves 3.6 GHz of UGBW and 57.8 ^∘^ of PM with all loading effect while consumes 10.5 mW from a 1.2-V supply. The second and third integrators adopt the same topology but with scaled bias currents, achieving UGBW of 4.7 and 3.3 GHz and PM of 58.0 and 57.8 ^∘^ while consuming 4.3 and 17.3 mW, respectively.

### Quantizer and DAC

**Fig. 5:**
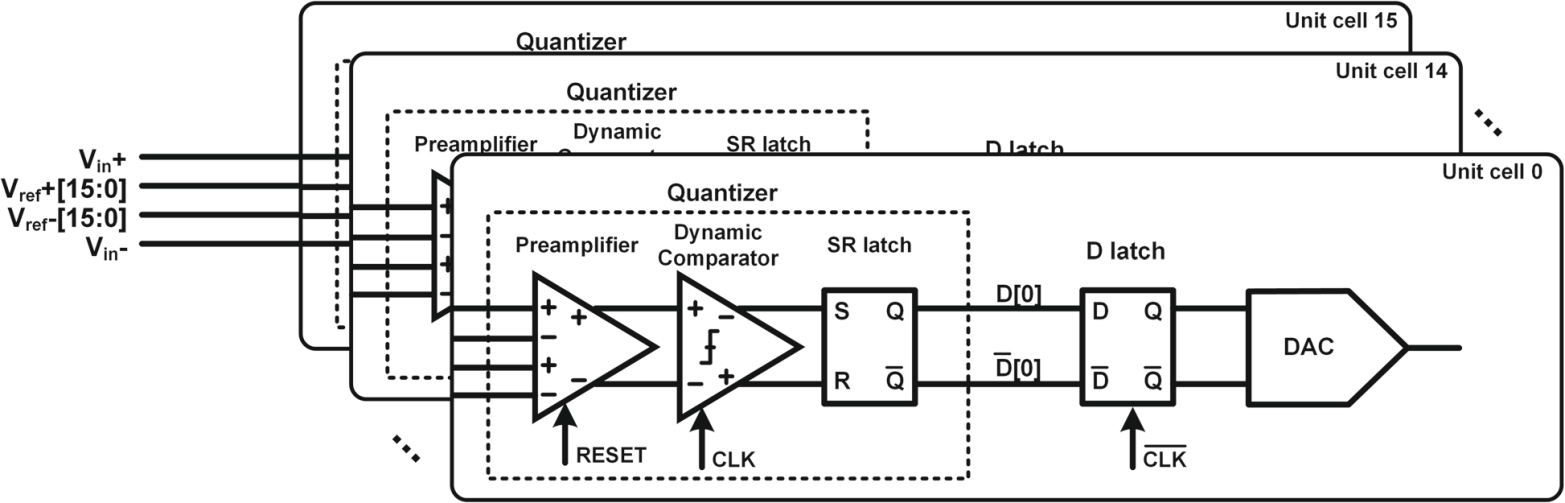
The simplified schematic of the quantizers and the DACs

As the schematic of the quantizer and DAC shown in Fig. 5, each consists of 16 unit cells. The quantizer is realized as a 4-bit flash ADCs with 16-level encoder generated from a 17-tap resistive ladder. The quantizer, whose operation duration is demanded by ELD to less than half a sampling period to ensure loop stability, is a key block as a limitation of maximum BW.

**Fig. 6:**
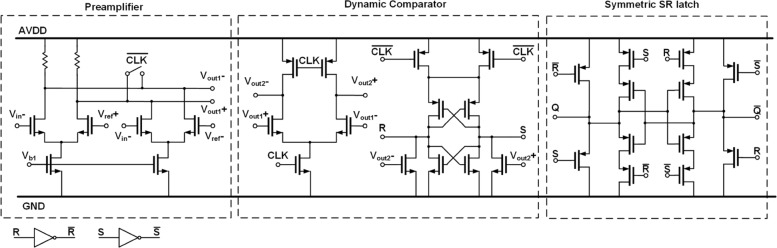
Transistor-level circuit of one unit element of the proposed quantizer

**Fig. 7:**
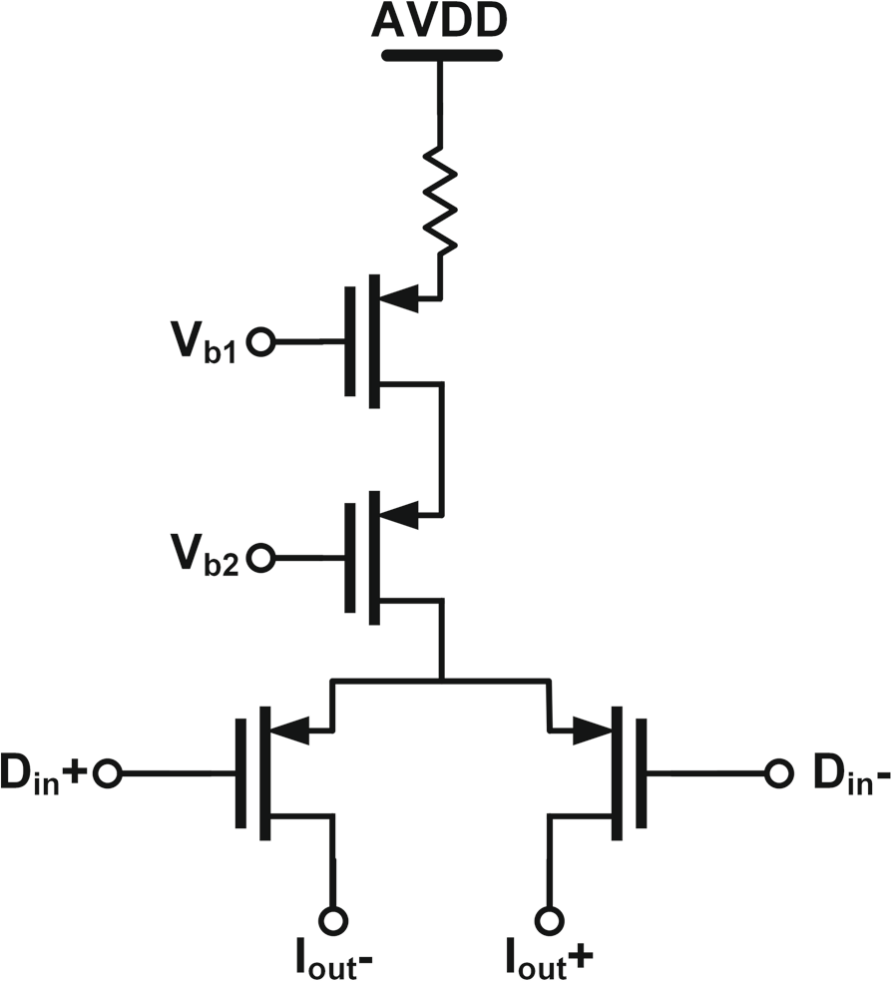
The PMOS current steering DAC unit element

To implement high-speed flash ADCs, a three-stage comparator architecture consisting of a preamplifier stage, a dynamic comparator stage, and a symmetric set-and-reset (SR) latch[12], illustrated as Fig. 6, is employed. The preamplifier for input-referred offset reduction is two resistively load differential pairs with a reset switch connecting across outputs to enable quick recovery. Unlike conventional dynamic comparators, the differential pair and cross-coupled inverters are split into two parts to minimize the amount of transistor in every current path for low-voltage supplies. When the clock turns to the high level, dynamic comparators start to make the input-dependent comparison decision. Then, the two outputs of each dynamic comparator are both reset to 0 as the clock return goes from high to low, triggering the regeneration and latching of the symmetric SR latch. Since only one transistor in each branch is active, the symmetric SR latch structure leads a strong loading driving capability. It allows a small transistor size with significant switch off speed and low power consumption. Furthermore, it results in equal delays of both output signals. The D latches before DAC units are low-level-sensitive with respect to the level of the clock signal, ensuring a half ELD duration. The transistor-level circuit of the current steering DAC unit is shown in Fig. 7.

## Results and Discussion

**Fig. 8:**
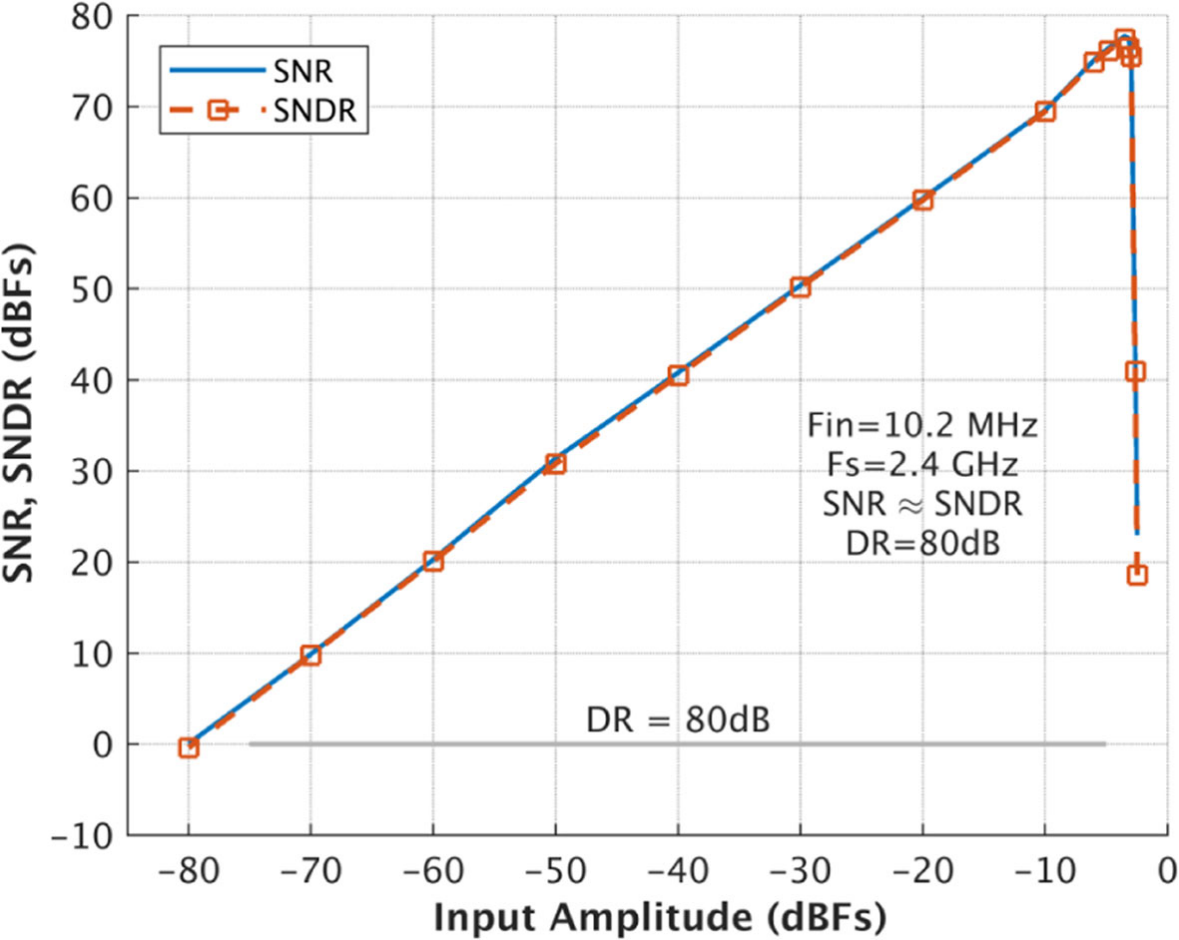
Post-simulated SNR and SNDR vs. input signal amplitude with a 10.2-MHz input

**Fig. 9:**
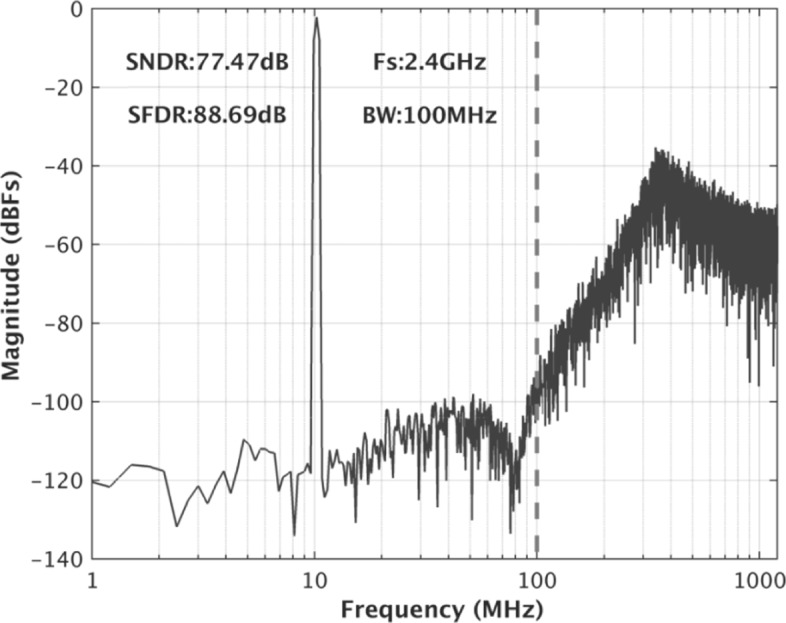
The post-simulated spectra with a single-tone input at 10.2 MHz

**Fig. 10:**
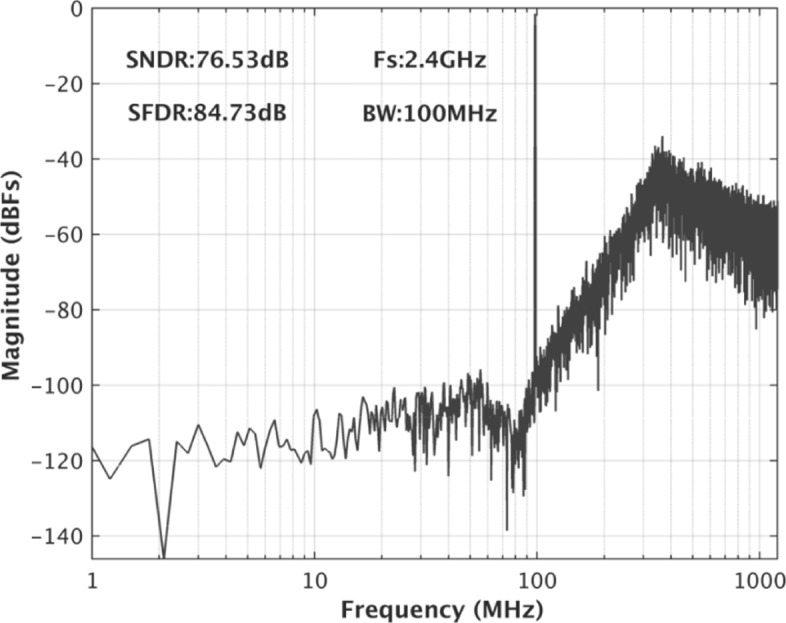
The post-simulated spectra with a single-tone input at 97 MHz

**Fig. 11:**
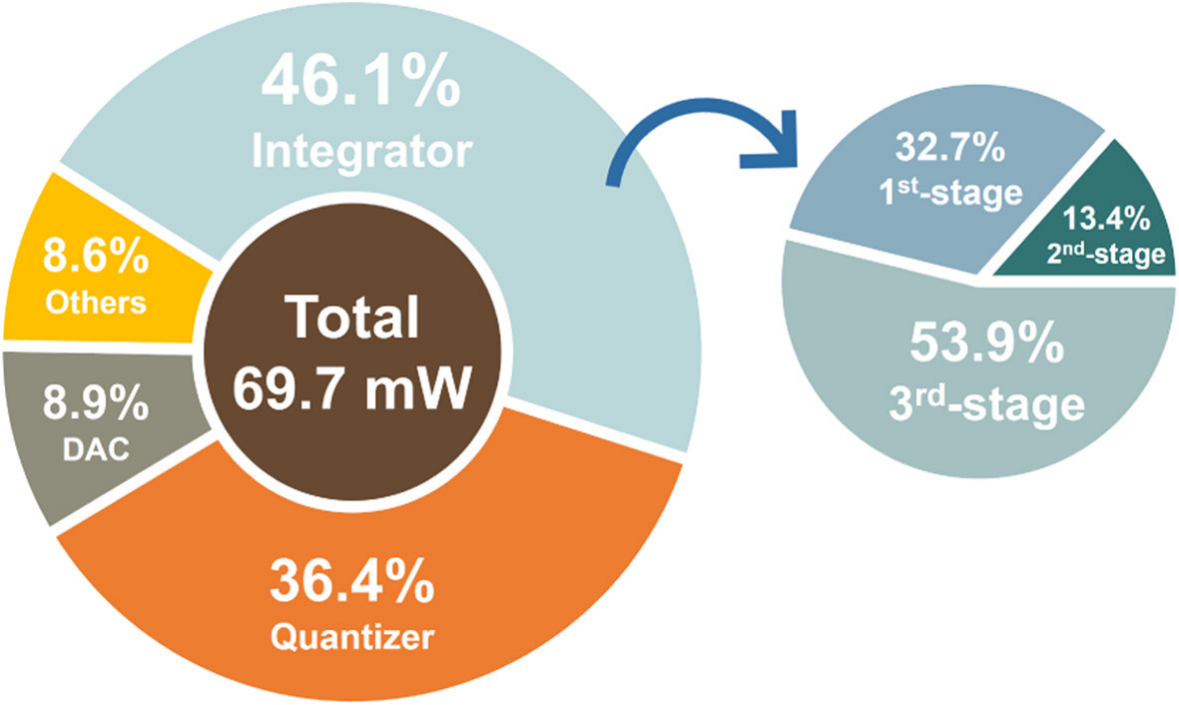
The post-simulated power consumption breakdown

The prototype *ΔΣ* modulator is built in a 40-nm CMOS process. As the post-simulated results of the SNR and SDNR vs. input amplitude at 10.2 MHz shown in Fig. 8, a 80-dB DR is achieved. Figures 9 and 10 show the simulated spectra with a − 3.52-dBF single-tone input at 10.2 MHz and 97 MHz, respectively, since 0 dBF corresponds to the 2.4 Vpp modulator full scale. The SNDR is 77.47 dB and 76.53 dB, respectively. As the breakdown consumption shown in Fig. 11, the modulator costs 69.7 mW power consumption. The integrator, the quantizer, and the DAC respectively consume 32.1 mW, 25.4 mW, and 6.2 mW. 6.0 mW power is consumed by the other currents including clock buffers, current biases, and the voltage references. The modulator achieves a Schreier FOM of 171.6 dB based on DR. Table 1 compares this work with several previously published works. The proposed modulator achieves wide BW with the highest FOM.

**Table 1:** Comparison of this work with the recent state-of-the-art *ΔΣ* modulator

Publication	This work	[8]	[9]	[3]	[7]
	Post-simulation result	Measured result	Measured result	Measured result	Measured result
**Process (nm)**	**40**	28	20	45	16
**Fs (GHz)**	**2.4**	8.0	2.184	4	2.88
**BW (MHz)**	**100**	465	80	125	160
**Peak SNR (dB)**	**77.74**	68	70	65.5	68.13
**Peak SNDR (dB)**	**77.47**	67	67.5	65	65.33
**DR (dB)**	**80**	72	73	70	72.1
**Power (mW)**	**69.7**	890	23	260	40
**FOM (dB)**	**171.6**	159	168	156.8	168.12

FOM = DR + 10log_10_(BW/Power)

## Conclusion

In this work, we proposed a 3rd-order 4-bit CT *ΔΣ* modulator with a single-loop feedforward topology. This modulator is designed in a 40-nm CMOS process and achieves 80 dB DR over a 100-MHz BW at a clock rate of 2.4 GHz. The low-power dissipation amplifier construction leads a high-energy efficiency, and the modulator consumes 69.7 mw from 1.2 V power supply and achieves a Schreier FOM of 171.6 dB.

## Data Availability

All data generated or analyzed during this study are included in this published article.

## Abbreviations

ADC: Analog-to-digital converters
BW: Bandwidth
CT: Continuous-time
DR: Dynamic range
DT: Discrete-time
ELD: Excess-loop-delay
LTE-A: Long-Term-Evolution Advanced
OSR: Oversampling ratios
PM: Phase margin
UGBW: Unity-gain bandwidth
